# Development and application of equilibrium optimizer for optimal power flow calculation of power system

**DOI:** 10.1007/s10489-022-03796-7

**Published:** 2022-07-18

**Authors:** Essam H. Houssein, Mohamed H. Hassan, Mohamed A. Mahdy, Salah Kamel

**Affiliations:** 1 Faculty of Computers and Information, Minia University, 61519 Minia, Egypt; 2 Ministry of Electricity and Renewable Energy, Cairo, Egypt; 3 Faculty of Computers and Artificial Intelligence, Beni-Suef University, Beni-Suef, Egypt; 4 Electrical Engineering Department, Faculty of Engineering, Aswan University, 81542 Aswan, Egypt

**Keywords:** Equilibrium optimizer, Meta-heuristics, CEC’20 test suite, Optimal power flow, Total fuel cost minimization, Total active power losses minimization

## Abstract

This paper proposes an enhanced version of Equilibrium Optimizer (EO) called (EEO) for solving global optimization and the optimal power flow (OPF) problems. The proposed EEO algorithm includes a new performance reinforcement strategy with the Lévy Flight mechanism. The algorithm addresses the shortcomings of the original Equilibrium Optimizer (EO) and aims to provide better solutions (than those provided by EO) to global optimization problems, especially OPF problems. The proposed EEO efficiency was confirmed by comparing its results on the ten functions of the CEC’20 test suite, to those of other algorithms, including high-performance algorithms, i.e., CMA-ES, IMODE, AGSK and LSHADE_cnEpSin. Moreover, the statistical significance of these results was validated by the Wilcoxon’s rank-sum test. After that, the proposed EEO was applied to solve the the OPF problem. The OPF is formulated as a nonlinear optimization problem with conflicting objectives and subjected to both equality and inequality constraints. The performance of this technique is deliberated and evaluated on the standard IEEE 30-bus test system for different objectives. The obtained results of the proposed EEO algorithm is compared to the original EO algorithm and those obtained using other techniques mentioned in the literature. These Simulation results revealed that the proposed algorithm provides better optimized solutions than 20 published methods and results as well as the original EO algorithm. The EEO superiority was demonstrated through six different cases, that involved the minimization of different objectives: fuel cost, fuel cost with valve-point loading effect, emission, total active power losses, voltage deviation, and voltage instability. Also, the comparison results indicate that EEO algorithm can provide a robust, high-quality feasible solutions for different OPF problems.

## Introduction

Achieving an optimal power flow (OPF) represents an essential and complex non-linear optimization problem in power systems [1]. From an optimization standpoint, the OPF problem involves minimization of an objective, such as voltage instability, fuel cost, and total emission. For this minimization, researchers have applied algorithms in order to obtain an optimized adjustment of control variables that respect constraints, i.e., equality and inequality of operating conditions [2]. These variables include the real power generator, voltages of generation buses, tap ratios of transformers, and reactive powers of shunt compensation capacitors [3].

Recently, several metaheuristic algorithms (MAs) [4] for solving the OPF problems have been proposed [5]. These include the adaptive multiple team perturbation-guiding Jaya (AMTPG-Jaya) algorithm [6], modified sine-cosine algorithm (MSCA) [7], modified grasshopper optimization algorithm (MGOA) [5], moth swarm algorithm (MSA) [8], electromagnetism mechanism algorithm (EM) [9], and colliding bodies optimization (CBO) [10]. Although these algorithms have solved the same kind of OPF problems, their objective functions, i.e., optimization goals, were different. Different goals lead to different optimized solutions and can therefore influence the optimization performance. Generally, optimization algorithm performance refers to the quality of the optimized solution and its computation time, i.e., time required for convergence of the algorithm. Although many MAs have provided satisfactory results, optimization problems have become increasingly challenging (as the number of optimized variables has increased), while satisfying many constraints and requirements. However, in spite of the advantages of algorithms, numerous existing meta-heuristic optimization algorithms do not constantly guarantee the globally optimum solution. In addition, owing to the variability of objectives Where, diverse functions are used for formulating the OPF problem, no algorithm can be considered the better-quality in solving all variants of OPF. Therefore, developing new meta-heuristic algorithms which can effectively handle diverse OPF formulations, is necessary [6]. Combining MAs - often referred to as hybridization- is effective in addressing the current optimization challenges [11]. Although hybridization improves optimization performance, it should be performed with adequate algorithms. Thus, selecting the algorithms is an important step. And common practice is to select them based on their standalone performance. Another way to improve algorithm performance is by adding optimization components to an original algorithm.

Therefore, to develop a more effective algorithm (than the methods typically employed) for solving OPF problems, we have studied recent algorithms and features. The Equilibrium Optimizer (EO) has attracted the attention of many researchers –in approximately one year, this method has been cited 500 times. In [12], the EO performed better than several other algorithms. This algorithm has been validated for over 58 benchmark functions, including composite functions and functions from the Congress On Evolutionary Computation 2017 (CEC’17), which are considered challenging optimization problems. To further validate its efficiency, the EO has also been applied to three canonical engineering problems, i.e., welded beam design, pressure vessel design, and tension/compression spring design. The EO optimized results yielded superior performance compared with those of seven other MAs. Although the EO has yielded promising results, the algorithm has some drawbacks. For example, depending on the optimization problem, slow convergence speed, convergence to a local minimum, performance dependence on algorithm parameters, and difficulty in achieving a balance between the exploration and exploitation phases, have been reported [13].

In this regard, the EO has gained much popularity in recent days in several fields of engineering and complex applications. Authors in [13] used EO in a similar context for solving combinatorial, global, engineering, and Multi-Objective problems. Authors in [14] applied Opposition Based Learning (OBL) at the initialization phase of EO for parameters identification of photovoltaic modules. In [15], authors combined the dimension learning hunting (DLH) with EO for multi-thresholding based COVID-19 CT images. Authors in [16], the support vector regression (SVR) method with equilibrium optimizer (EO) is combined for stock market prediction. In [17], authors developed a new variant of EO called general learning equilibrium optimizer (GLEO), they utilizes a general learning strategy to explore the promising regions, the GLEO is employed as a wrapper feature selection method, to select a subset of informative biological dataset’s features. Authors in [18] proposed an enhanced EO version using ReliefF algorithm and the local search strategy, the introduced feature selection algorithm, is tested on 16 UCI datasets and 10 biological datasets. In [19], authors introduced an adaptive variant of EO called LWMEO for solving the engineering design problems, the Lévy flight random walk is utilized to enhance the traditional EO exploration, and spiral encirclement mechanism to enhance the exploitation process. In [20], an improved variant of the EO (IEO), to optimize the optimal power flow (OPF) problem, the IEO uses the chaotic equilibrium pool to improve the information sharing between individuals. In [21], authors suggested an algorithm that combines the a modified version of the EO and the extreme learning machine (ELM). To enhance the exploratory search, a gaussian mutation method is incorporated to the original EO.

Some studies have focused on overcoming these shortcomings. Recently, the Lévy Flights (LF) algorithmic feature has yielded excellent results in improving MA performance [22], and consequently has attracted attention from optimization algorithm developers. Indeed, LF has been integrated into the algorithms of some MAs, such as Grey wolf optimizer (GWO) [23], Particle swarm optimization [24, 25], Evaporation rate water cycle algorithm [26], Whale Optimization Algorithm (WOA) [27], Chimp optimization algorithm [28], marine predators algorithm [29], and Lévy flight distribution [30]. The results have shown that (in general) LF improves standard MAs by strengthening the local search, escaping local minima, enhancing the convergence speed, or improving the exploration-exploitation balance. With these benefits in mind, incorporating LF features into an algorithm seems a promising avenue for addressing the aforementioned shortcomings. Therefore, the motivations of this paper are to:

1. Device a methodology for reinforcing the exploration and exploitation phases of MAs, thereby providing improved solutions to optimization problems;
2. Investigate the OPF and provide enhanced solutions;
3. Propose an optimization algorithm that exhibits better performance than recent and high-performance algorithms.

Thus, this paper proposes an enhanced EO (EEO), which includes an LF component and a new reinforcement strategy for solving OPF problems more efficiently than other methods. In particular, the proposed enhancing method consists of three components for improving the local and global searches. The aim is to reduce the potential weaknesses (such as premature convergence, unbalanced exploration and exploitation phases, and convergence to a local optimum) of the standard EO. Thus, the proposed algorithm aims to solve OPF problems more efficiently (than other methods) and to serve as a high-performance optimization tool [31]. To achieve this goal, the efficiency of the proposed EEO is evaluated on ten benchmark functions of the CEC’20 test suite. The proposed EEO is then used to solve the OPF problem of a standard IEEE 30-bus system. The fuel cost, fuel cost with value-point loading effect, emission, total active power losses, voltage stability enhancement, and voltage deviation are all individually optimized. Simulation results are compared with the results of the original EO, some of the most recent algorithms, and high-performance optimizers and winners of IEEE CEC competitions including; Moth-flame optimization algorithm (MFO), Sine Cosine Algorithm (SCA), Whale Optimization Algorithm (WOA), Grey wolf optimizer (GWO), Harris hawk optimization algorithm (HHO), Black Widow Optimization Algorithm (BWO), Evolution Strategy with Covariance Matrix Adaptation (CMA-ES), Ensemble Sinusoidal Differential Covariance Matrix Adaptation (LSHADE_cnEpSin), Improved Multi-Operator Differential Evolution algorithm (IMODE), Adaptive Gaining Sharing Knowledge (AGSK) and the original EO.

In summary, the features that distinguish our work from previous studies are as follows:

- We proposed an enhanced algorithm, i.e., the EEO, which includes a new exploitation-exploration method.
- The EEO performance is analyzed over 10 benchmark functions of the CEC’20 test suite. This benchmark is more recent than other related works. The optimized results are compared with the standard EO and other recently published algorithms.
- Statistical and qualitative analyses validate the performance of the proposed EEO.
- The OPF objective functions are more comprehensive than those considered in many other studies on the topic. That is, six functions (fuel cost, fuel cost with value-point loading, emissions, total real power losses, voltage instability, and voltage deviation) are minimized.
- The proposed EEO achieved high-quality optimized solutions for the standard IEEE 30-bus power system compared with the results of seven recent algorithms and 20 published results.

The rest of this paper are organized as follows: Section 2 presents some preliminaries about EO and other used enhancement methods i.e., the original EO, the LF method. Section 3 presents the details of the proposed EEO algorithm, and its components. Section 4 introduces the formulation of OPF problem, i.e., the mathematical model and constraints. Section 5 presents the results obtained and analyses performed by the proposed EEO and competitive algorithms on the CEC’20 test suite and the OPF problems. Section 6 concludes the paper.

## Preliminaries

The different components of the proposed algorithm, i.e., the original EO, the LF feature, are described in the following subsections.

### Equilibrium optimizer (EO)

Inspired by physics observations, the authors of [12] have proposed an EO. Specifically, EO is based on the physics laws governing the balance of concentrations of nonreactive constituents in a controlled volume. An equation defines the conservation of mass that enters and leaves a specific volume and the system always tends to an equilibrium point. The EO algorithm is based on the ability to reach this point. Indeed, the algorithm tries to stabilize the concentration within the system. The three main mathematical steps of EO are: *1)* Initialization, *2)* Equilibrium pool and candidates, and *3)* Concentration update (we describe these steps below and refer readers to [12] for additional details about the EO).

#### Initialization

Similar to MAs based on population evolution, EO generates a population randomly. The population consists of particles and a uniform distribution is obtained. Particles are defined by concentration vectors. The initial population is generated from:

1 $$ P_{i}^{\text{initial}}=P_{\min }+\operatorname{rand}_{i}\left( P_{\max }-P_{\min }\right) \quad i=1,2, \ldots, n $$

Where, $P_{i}^{\text {initial}}$ is the vector corresponding to the initial concentration of particle *i*, $P_{{\max \limits } }$ and $P_{{\min \limits } }$ are the upper and lower bounds, respectively, *n* is the number of particles in the population, and rand_*i*_ generates a random value ∈ [0,1].

#### Equilibrium pool and candidates

From an optimization standpoint, EO employs a pool of five particles to achieve the unknown state of equilibrium, which represents the optimal solution. The pool is composed of the four best-so-far particles for diversification purposes. The average of these four particles is employed for the exploitation process. The pool is defined as:

2 $$  \overrightarrow{\mathrm{P}}_{\text{eq}}=\left[\overrightarrow{\mathrm{P}}_{\text{eq}(1)}, \overrightarrow{\mathrm{P}}_{\text{eq}(2)}, \overrightarrow{\mathrm{P}}_{\text{eq}(3)}, \overrightarrow{\mathrm{P}}_{\text{eq}(4)}, \overrightarrow{\mathrm{P}}_{\text{eq}(\text{avg})}\right]. $$

#### Concentration update

At each iteration, the EO updates the particle population through the following equation:

3 $$ \vec{P}=\overrightarrow{P_{\text{eq}}}+(\vec{P}-\overrightarrow{P_{\text{eq}}}) \vec{F}+\frac{\vec{R}}{\vec{\lambda}} (1-\vec{F}), $$

Where, $\vec {F}$ influences the exploration-exploitation balance and is defined as follows:

4 $$ \vec{F}=e^{-\vec{\lambda}\left( t-t_{0}\right)}, $$

Where, *λ* is a random value ∈ [0,1], and and the value of t decreases with increasing iteration number *iter*, as follows,

5 $$ t=\left( 1-\frac{iter}{Max\_{iter }}\right)^{\left( a_{2} \left( \frac{iter}{Max\_{iter }}\right)\right)}. $$

*iter* is the current iteration and *Max*_*iter* is the maximum number of iterations. The constant *a*_2_ controls the exploitation; as *a*_2_ increases, the intensification process improves, but the exploration capability decreases. The vector $\vec {t}_{0}$ is computed as follows:

6 $$ \vec{t}_{0}=\frac{1}{\vec{\lambda}} \ln \left( -a_{1} \operatorname{sign}(\vec{r}-0.5)\left[1-e^{-\vec{\lambda} t}\right]\right)+t, $$

Where, the constant *a*_1_ controls the diversification. The term sign(*r* − 0.5) designates the diversification and intensification directions. The exploration ability increases with increasing *a*_1_ value, but the exploitation capability decreases. The vector $\vec {R}$ is referred to as the generation rate and is computed as follows:

7 $$ \vec{R}=\overrightarrow{\text{RCP}} (\overrightarrow{P_{\text{eq}}}-\vec{\lambda} \vec{P}) e^{-\vec{\lambda} \left( t-t_{0}\right)}, $$

Where, $\overrightarrow {\text {RCP}}$ is :

8 $$ \overrightarrow{\text{RCP}}=\left\{\begin{array}{ll} 0.5 r_{1} & r_{2}>\text{RP} \\ 0 & \text{ otherwise }, \end{array}\right. $$

Where, *r*_1_ and *r*_2_ are random values ∈ [0,1] and RP is a variable that also influences the exploitation-exploration balance.

## The proposed EEO algorithm

### Initialization

The proposed EEO integrates the above-mentioned strategies. Particularly, the EEO initializes its population with LF distribution as follows:

9 $$ P_{i}^{\text{initial}}=P_{\min }+\operatorname{Levy}\left( \beta\right) \times \left( P_{\max }-P_{\min }\right) \quad i=1,2, {\ldots} {\ldots} NP $$

Where, $P^{\text {initial}}_{i}$ are the initial values of the *i*^*th*^ particle, $P_{{\min \limits } }$ and $P_{{\max \limits } }$ are the lower and upper bounds, respectively. $\operatorname {Levy}\left (\beta \right )$ is LF random walk described. Previous work has confirmed that LF allows effective coverage of the search region, and hence candidate solutions will most likely converge to (near)-optimal solutions. Indeed, a previous study [32] has demonstrated that LF is an effective mechanism for escaping regions with local minima (even those with many deep local minima).

### Reinforce exploration

During the exploration stage, algorithm particles search the problem space broadly in order to identify promising areas. The exploration performed by the original EO algorithm involves only searches near the best particle, i.e., *P*_*eq*_, and can therefore be improved. Thus, this work proposes a new reinforcement exploration method that mutates the search particles by selecting two particles in the population as follows:

1. Based on the fitness value, the population is divided into two parts, i.e., the best and worst solutions;
2. The *P*_*r*1_ solution from the best fitness solutions and *P*_*r*2_ from the worst fitness solutions are selected via the tournament selection method, which is defined by:
  10 $$ Pt_{j} = Peq_{j} + (P_{r2}(j)- P_{i,j}) * x+ (P_{i,j}- P_{r1}(j)) * y. $$
  Where, x, y operators retain the stochastic nature and define the convergence direction of search particles, when generating *Pt* solution; x and y are generated randomly through the following equations:
  11 $$ x = 0.05 + 0.95*rand $$
  12 $$ y = 0.9 + 0.1*rand $$
  Where, *rand* is a random number generated within average ∈ [0,1].

### Reinforce exploitation

During the exploitation process, the particles are crowded for searching around the identified promising areas from early exploration. As illustrated below, search-particle evolution is achieved by minimizing the distance from the best agent *P*_*eq*_. (3) of the original EO algorithm is slightly adapted in the proposed EEO and is given as follows:

13 $$ P_{m}(j) = P(i,j) + \vec{F}(P_{i,j} - Peq(j)) + (\vec{G}/\vec{\lambda})(1-\vec{F}) $$

Where, vectors *F* and *λ* are defined by (4) and (3) respectively, and *Peq*(*j*) refers to the j-th portion of the best particle, *P*(*i*,*j*) refers the j-th portion of the i-th particle.

### Balancing exploration and exploitation

During the first iterations, strengthening the exploration phase is essential for algorithm identification of promising areas in the problem space, while the subsequent iterations exploit the identified areas. Therefore, the following strategy is proposed for balancing the exploration and exploitation phases:

14 $$ P(new)_{j} =\left\{\begin{array}{ll} P_{m}(j) & \text{if } \left( \text{rand} > 0.6\right)\\ Pt_{j} & \text{else if }\left( z > 1/2\right) \\ P_{i,j} & otherwise \end{array}\right. $$

Where, *z* is computed as follow:

15 $$ z=\left( 1-\frac{iter}{Max\_{iter }}\right)e^{\left( a_{2} \left( \frac{iter}{Max\_{iter }}\right)\right)}. $$

The value of *z* decreases with increasing iteration number *iter*; *iter* is the current iteration and *Max*_*iter* is the maximum number of iterations. The variable *a*2 is a constant that controls the exploitation step. During the optimization process, the particles evolve by mutating some parts of each particle and keeping the best information parts; indeed, (14) retains the best parts of each particle *P*_*i*,*j*_.

Algorithm 1 presents the pseudo-code of the EEO algorithm, Where, *iter* refers to the current iteration number and *Max*_*iter* refers to the total number of iterations.

**Figure:**
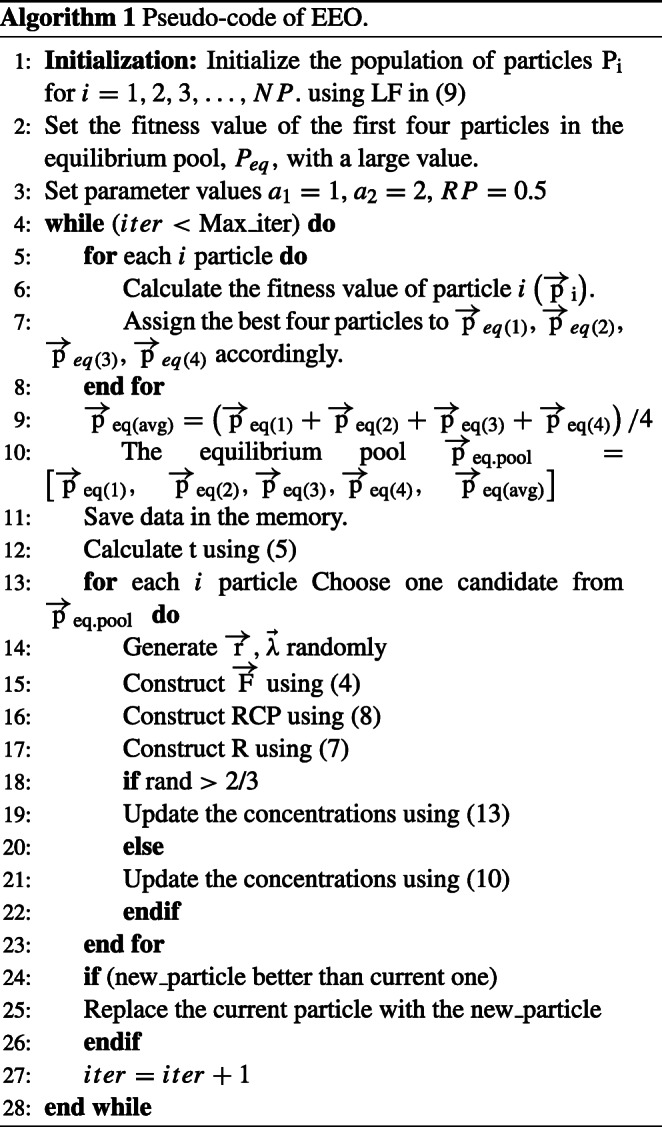

### Computation of complexity

The time complexity of the EEO relies mainly on the process of updating solutions’ positions. Therefore, it can be formulated as follows:

The time complexity is mainly depends on the number of particles (NP), the optimization problem dimension (D), the total number of iterations (T) and the function evaluations’s cost (C). In a particular way, the time complexity of EEO is computed as follow: O(EEO) = O ((problem definition) + (initialization) + O (cost function) + O(Solution update)), consequently,

16 $$ O(EEO) = O (1 + NP*D + T*NP*C + T*NP*D) $$

The terms of each component in (16) can be defined as below:

- The problem definitions require O(1) time.
- The particles initialization step require O(NP×D) time.
- Evaluation of the population particles demands O(T × C× NP) time.
- The population particles update phase require O(T × NP × D) time.

Hence, the overall EEO time complexity in polynomial order.

## OPF problems - mathematical formulation

### General structure of OPF

We modeled the OPF problem as follows [33]:

17 $$ Minimize: f(x,u) $$

subject to:

18 $$ g\left( x,u\right)=\mathrm{0} $$

19 $$ h\left( x,u\right)<=\mathrm{0} $$

Where, *f* is the objective functions, *g* and *h* are sets of equality constraints and inequality constraints of the power system network, which are voltage and angle of load buses respectively. And *x*, which represents various state variables, is defined as follows:

20 $$ x=[P_{G_{1}},V_{L_{1}}, \ldots, V_{L_{NL}},Q_{G_{1}}, \ldots, Q_{G_{NG}},S_{l_{1}}, \ldots, S_{l_{nl}}] $$

Where, $P_{G_{1}}$ is the active power of the generator at the slack bus, $V_{L_{i}}$ is the voltage magnitude of the *i*^*th*^ load bus, $Q_{G_{i}}$ is the reactive power output of the *i*^*th*^ generator, and $S_{l_{j}}$ is the line loading of the *j*^*th*^ line. *NL* is the number of load buses. *NG* is the number of generators and *ln* is the number of transmission lines. Furthermore, *u*, the vector of control variables, which are included real and reactive power outputs from generators, bus voltages, series and/or shunt capacitors (reactors), tap-changer transformers setting defined as [33]:

21 $$ u=[P_{G_{2}}, \ldots, P_{G_{NG}},V_{G_{1}}, \ldots,V_{G_{NG}},Q_{c_{1}}, {\ldots} ,Q_{c_{Nc}},T_{1}, \ldots,T_{NT}] $$

Where, $P_{G_{i}}$ is the *i*^*th*^ bus generator real power excluding the swing generator, $V_{G_{i}}$ is the voltage magnitude of the *i*^*th*^ generator, $Q_{c_{d}}$ is the shunt compensation of the *d*^*th*^ bus, *T*_*k*_ is the *k*^*th*^ branch transformer tap, *NT* is the number of regulating transformers, and *NC* is the number of shunt compensators. Any value within its range can be assumed as a control variable.

### Objective functions for the OPF

The proposed EEO performance is evaluated over six case studies with various objective functions for a standard IEEE 30-bus system [33].

#### Case 1: fuel cost minimization

We can relate the total fuel cost ($/h) to the generated power (MW) as follows:

22 $$ f(x,u) = FC = \sum\limits_{i=1}^{NG} (a_{i} + b_{i} P_{Gi} + c_{i} P^{2}_{{Gi}}), $$

Where, *a*_*i*_, *b*_*i*_, and *c*_*i*_ aare the cost coefficients of the thermal generators $P_{{G}_{i}}$.

#### Case 2: fuel cost with value-point loading effect minimization

Minimization of the total fuel cost with value-point loading effect is achieved through the following relation:

23 $$ \begin{array}{@{}rcl@{}} f(x,u) = FC_{vp} &=& \sum\limits_{i=1}^{NG} (a_{i} + b_{i}P_{{G}_{i}}+c_{i}P^{2}_{Gi}\\&&+\mid d_{i} \times \sin (e_{i} \!\times\! (P^{min}_{Gi} - P_{{G}_{i}})) \mid ) \end{array} $$

Where, *d*_*i*_ and *e*_*i*_ are the cost coefficients of the *i*^*th*^ thermal generators. Table 1 lists the coefficient values presented in [34].

**Table 1:** Cost coefficients of the thermal power generators

Generator	Bus	*a*	*b*	*c*	*d*	*e*
G1	1	0	2	0.00375	18	0.037
G2	2	0	1.75	0.0175	16	0.038
G3	5	0	1	0.0625	14	0.04
G4	8	0	3.25	0.00834	12	0.045
G5	11	0	3	0.025	13	0.042
G6	13	0	3	0.025	13.5	0.041

#### Case 3: emission minimization

The third objective function is used to minimize the emission produced by the thermal generation units:

24 $$ f(x,u) = E={\sum}_{i=1}^{NG} (0.01 \times (\alpha_{i}+\beta_{i}P_{{G}_{i}}+\gamma_{i}P^{2}_{Gi})+ \omega_{i}e^{\mu_{i}P_{Gi}} ) $$

Where, *α*_*i*_, *β*_*i*_, *γ*_*i*_, *ω*_*i*_, *μ* are the emission coefficients of the thermal generators. The values presented in [34] are listed in Table 2.

**Table 2:** Emission coefficients of the thermal power generators

Generator	Bus	*α*	*β*	*γ*	*ω*	*μ*
G1	1	4.091	-5.554	6.49	0.0002	2.857
G2	2	2.543	-6.047	5.638	0.0005	3.333
G3	5	4.258	-5.094	4.586	0.000001	8
G4	8	5.326	-3.55	3.38	0.002	2
G5	11	4.258	-5.094	4.586	0.000001	8
G6	13	6.131	-5.555	5.151	0.00001	6.667

#### Case 4: total real power losses minimization

The fourth objective minimizes the total real power loss and is expressed as follows:

25 $$ f(x,u) = P_{loss}=\sum\limits_{q=1}^{nl} (G_{q_{(ij)}}\times ({V^{2}_{i}}+{V^{2}_{j}}-2V_{i}V_{j}\cos(\delta_{ij} ))) $$

Where, $G_{q_{(ij)}}$ is the conductance transfer of branch *q*_(*ij*)_ and *δ*_*ij*_ is the difference in voltage angles. *V*_*i*_ is the voltage at bus *i* and *V*_*j*_ is the voltage at bus *j*.

#### Case 5: voltage instability minimization

The power system stability refers to the ability of the system to maintain bus voltages within admissible limits. The voltage stability index (L-index) of each bus, which is an accepted metric for assessing this stability, is given as:

26 $$ L_{j}=\mid 1-\sum\limits_{i=1}^{NG} (F_{ji}\frac{V_{i}}{V_{j}})\mid $$

Where, *F*_*ji*_ = −[*Y*_*LL*_]^− 1^[*Y*_*LG*_]. *Y*_*LG*_ and *Y*_*LL*_ are submatrices of the admittance matrix at a specific bus. Thus, we can express the voltage stability as follows [35]:

27 $$ f(x,u) = L_{max}= max[L_{j}], \text{Where,} j=1,2,3,\ldots,NL $$

Where, *L*_*j*_ is the L-index of the *j*^*th*^ load bus.

#### Case 6: voltage deviation minimization

The last objective function is used to minimize the cumulative deviation of voltages obtained for the entire load bus:

28 $$ f(x,u) = VD= \sum\limits_{i=1}^{NG} \mid V_{L_{p}}-1 \mid $$

### Constraints

Usually, the OPF constraints are split into two categories: *i)* equality constraint and *ii)* inequality constraints [5]. The equality constraints are as follows:

29 $$ P_{Gi} - P_{Di}= V_{i}\sum\limits_{j=1}^{NB} V_{j}(G_{ij} \cos \theta_{ij}+B_{ij} \sin \theta_{ij}) $$

30 $$ Q_{Gi} - Q_{Di}= V_{i}\sum\limits_{j=1}^{NB} V_{j}(G_{ij} \sin \theta_{ij}+B_{ij} \cos \theta_{ij}) $$

Where, *𝜃*_*ij*_ is the difference in voltage angles. *P*_*Di*_ and *Q*_*Di*_ are the active load demand and the reactive load demand, respectively. *G*_*ij*_ is the transfer conductance and *B*_*ij*_ is the susceptance.

The inequality constraints, which are associated with five parts of the power system, are given as follows:

- Generator constraints:
  31 $$  V_{G_{i}}^{\min} \leq V_{G_{i}} \leq V_{G_{i}}^{\max}, i=1,\ldots,NG $$
  32 $$  P_{G_{i}}^{\min} \leq P_{G_{i}} \leq P_{G_{i}}^{\max}, i=1,\ldots,NG $$
  33 $$  Q_{G_{i}}^{\min} \leq Q_{G_{i}} \leq Q_{G_{i}}^{\max}, i=1,\ldots,NG $$
- Transformer tap setting constraints
  34 $$  T_{j}^{\min} \leq T_{j} \leq T_{j}^{\max}, j=1, {\ldots} ,NT $$
- Shunt compensator constraints
  35 $$ Q_{C_{d}}^{\min} \leq Q_{C_{d}} \leq Q_{C_{d}}^{\max}, d=1,\ldots,NC $$
- Voltages at load bus constraints
  36 $$  V_{L_{h}}^{\min} \leq V_{L_{h}} \leq V_{L_{h}}^{\max}, h=1,\ldots,NL $$
- Transmission line loading constraints
  37 $$  S_{l_{k}}\leq S_{l_{k}}^{\max}, k=1,\ldots,nl. $$

We use the following penalty function to ensure feasible solutions, where all the constraints are respected:

38 $$ \begin{array}{@{}rcl@{}} penalty&=&K_{p}(P_{G_{1}}-P_{G_{1}}^{Lim})^{2}+K_{q} \sum\limits_{i=1}^{NG} (Q_{G_{i}}-Q_{G_{i}}^{Lim})^{2}\\ && +K_{v} \sum\limits_{i=1}^{NL} (V_{L_{i}}-V_{L_{i}}^{Lim})^{2}+K_{s} \sum\limits_{i=1}^{nl} (S_{l_{i}}-S_{l_{i}}^{Lim})^{2} \end{array} $$

**Fig. 1:**
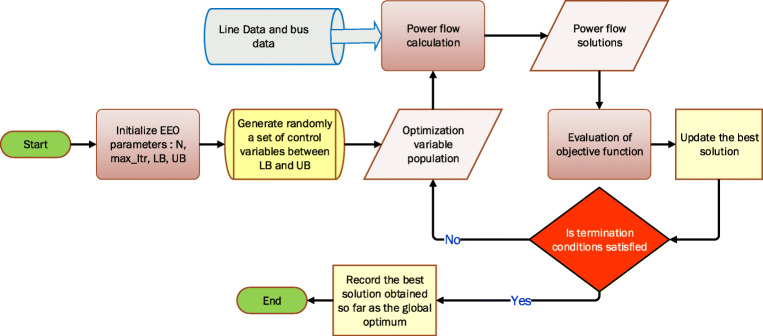
Flowchart of EEO for the OPF problem

Where, *K*_*p*_, *K*_*q*_, *K*_*v*_, and *K*_*s*_ are penalty factors. In this study, *K*_*p*_ = *K*_*q*_ = *K*_*v*_ = 100, and *K*_*s*_ = 100,000. Figure 1 shows the flowchart of the OPF optimization performed by the proposed EEO.

## Simulations results and discussion

Before applying the proposed algorithm to OPF problems, we assess the proposed EEO efficiency on the IEEE Congress on Evolutionary Computation 2020 (CEC’20) [36]. The simulation results of the proposed EEO are compared with those obtained from various classes of existing optimization methodsincluding; LSHADE_cnEpSin, CMA-ES, IMODE, AGSK, MFO, SCA, WOA, GWO, HHO, BWO, and EO.

### Parameter settings

To conduct a fair comparison, the EEO algorithm and the other competitors are investigated through 30 runs. The function evaluations (FEs) number is set to 150,000 for all considered problems. Table 3 shows the parameters’ setting for each algorithm. The parameters of the proposed algorithm and competitive algorithms are tuned first. In order to get the suitable parameter values corresponding to the best performance when applying the test methods, the taguchi robust design parameter is used. The Taguchi [37] method utilizes the Orthogonal Array (OA) and the mean analysis to investigate the effects of the algorithm’s parameters based on the statistical analysis of experiments. The OA is a fractional factorial matrix of numbers arranged so that each row represents the level of the factors in each run and each column represents a specific factor that can be changed from each run.

**Table 3:** Parametrization of EEO and the other algorithms

Algorithms	Parameters setting
Common Settings	Population size: *NP* = 30
	Maximum iterations: *Max*_*iter*_ = 5, 000
	Number of independent runs : 30
LSHADE_cnEpSin	$H=5, NP_{\min \limits }= 4 , Pbest \ rate = 0.11, Arc \ rate = 1.4, ps = 0.5, pc = 0.4$
CMA-ES	*alpha*_*mu*_ = 1.2020
AGSK	$ KF_{p}ool = [0.1, 1.0, 0.5, 1.0] , NP_{\min \limits }= 12$
	*KR*_*pool* = [0.2, 0.1, 0.9, 0.9]
IMODE	*arch*_*r*_*ate* = 2.6
MFO	*b* = 1
SCA	*A* = 1.57
WOA	*α* = 1
HHO	*E*0 = 1.67,*E*1 = 1
BWO	Percent of Crossover = 0.56 and Percent of Mutation = 0.78
EO	*a*_1_ = 0.778,*a*_2_ = 0.556
EEO	*a*_1_ = 2.33,*a*_2_ = 1.89
	*x* = [0.07, 0.50],*y* = [0.45, 0.89]

### Experimental series 1: applying EEO for solving CEC’20 test suit

#### Statistical results and analysis

The algorithm results on the CEC’20 functions are compared. In particular, the efficiency of each algorithm is measured with the average of the best solutions obtained at each run and the corresponding standard deviation (STD). Table 4 presents the average and STD values of each algorithm for functions of 10-dimension, i.e., *Dim* = 10. The best results are shown in boldface.

**Table 4:** Average and STD obtained by the algorithms on the CEC’20 test suite with *Dim* = 10

F	Measure	LSHADE_cnEpSin	IMODE	AGSK	CMA-ES	MFO	SCA	WOA	GWO	HHO	BWO	EO	EEO
F1	Average	**1.00E + 02**	**1.00E + 02**	**1.00E + 02**	9.69E + 08	1.46E + 08	4.95E + 08	1.03E + 04	4.63E + 07	2.01E + 05	1.38E + 09	1.72E + 07	**1.00E + 02**
	STD	1.87E-14	0.00E + 00	7.29E-15	1.25E + 09	4.13E + 08	1.81E + 08	1.72E + 04	1.33E + 08	8.95E + 04	8.28E + 08	7.69E + 07	4.61E-15
F2	Average	**1.18E + 03**	**1.18E + 03**	1.30E + 03	2.33E + 03	2.00E + 03	2.15E + 03	1.93E + 03	1.50E + 03	2.01E + 03	1.82E + 03	1.94E + 03	1.26E + 03
	STD	8.58E + 01	5.98E + 01	9.95E + 01	4.38E + 02	3.73E + 02	1.59E + 02	3.30E + 02	2.59E + 02	3.70E + 02	2.46E + 02	3.51E + 02	9.70E + 01
F3	Average	**7.14E + 02**	7.17E + 02	7.16E + 02	7.31E + 02	7.38E + 02	7.65E + 02	7.72E + 02	7.26E + 02	7.76E + 02	7.27E + 02	7.73E + 02	**7.14E + 02**
	STD	1.99E + 00	1.83E + 00	2.64E + 00	4.99E + 00	1.17E + 01	5.15E + 00	1.70E + 01	7.84E + 00	1.13E + 01	8.00E + 00	2.40E + 01	1.42E + 00
F4	Average	**1.90E + 03**	**1.90E + 03**	**1.90E + 03**	2.07E + 03	**1.90E + 03**	1.91E + 03	**1.90E + 03**	**1.90E + 03**	1.91E + 03	5.07E + 03	1.97E + 03	**1.90E + 03**
	STD	1.60E-01	3.71E-01	2.49E-01	6.98E + 02	5.81E + 00	4.79E + 00	1.73E + 00	1.86E + 00	2.83E + 00	4.09E + 03	2.08E + 02	1.21E-01
F5	Average	1.97E + 03	1.76E + 03	**1.72E + 03**	7.61E + 04	2.69E + 04	2.16E + 04	7.74E + 04	5.22E + 03	3.05E + 04	2.52E + 05	3.89E + 04	**1.72E + 03**
	STD	1.44E + 02	6.26E + 01	3.75E + 01	5.46E + 04	3.18E + 04	4.17E + 04	1.24E + 05	3.04E + 03	4.28E + 04	2.24E + 05	1.04E + 05	7.93E + 00
F6	Average	1.60E + 03	1.60E + 03	1.60E + 03	1.60E + 03	1.60E + 03	1.60E + 03	1.60E + 03	1.60E + 03	1.62E + 03	1.61E + 03	1.62E + 03	1.60E + 03
	STD	1.98E-01	1.14E-01	0.00E + 00	9.26E-01	3.59E + 00	2.85E + 00	3.66E + 00	5.17E + 00	2.00E + 01	7.74E + 00	3.05E + 01	1.57E-01
F7	Average	2.15E + 03	2.11E + 03	**2.10E + 03**	7.26E + 03	1.13E + 04	6.70E + 03	1.61E + 04	7.93E + 03	1.21E + 04	2.98E + 04	2.78E + 03	**2.10E + 03**
	STD	6.27E + 01	1.41E + 01	2.89E-01	4.64E + 03	1.10E + 04	3.21E + 03	1.11E + 04	4.80E + 03	9.70E + 03	6.40E + 04	1.07E + 03	3.26E + 00
F8	Average	2.30E + 03	2.30E + 03	**2.29E + 03**	2.34E + 03	2.30E + 03	2.35E + 03	2.37E + 03	2.33E + 03	2.31E + 03	2.37E + 03	2.36E + 03	**2.28E + 03**
	STD	1.88E + 01	1.35E + 01	2.97E + 01	6.91E + 01	1.10E + 01	2.08E + 01	2.84E + 02	1.01E + 02	4.42E + 00	3.05E + 01	8.27E + 01	3.69E + 01
F	Average	2.73E + 03	**2.54E + 03**	**2.57E + 03**	2.70E + 03	2.77E + 03	2.73E + 03	2.78E + 03	2.74E + 03	2.82E + 03	2.75E + 03	2.76E + 03	2.66E + 03
	STD	2.81E + 00	8.65E + 01	1.12E + 02	3.53E + 01	1.07E + 01	9.22E + 01	1.85E + 01	7.76E + 00	1.17E + 02	7.28E + 01	1.14E + 02	1.09E + 02
F10	Average	2.93E + 03	2.92E + 03	**2.88E + 03**	2.97E + 03	2.94E + 03	2.95E + 03	2.94E + 03	2.94E + 03	2.92E + 03	2.97E + 03	2.95E + 03	**2.91E + 03**
	STD	2.05E + 01	2.28E + 01	6.66E + 01	6.65E + 01	3.83E + 01	1.60E + 01	3.01E + 01	1.70E + 01	4.99E + 01	1.90E + 01	5.70E + 01	2.13E + 01
Friedman mean rank		3.25	2.35	2.05	9.10	7.90	8.20	8.50	6.40	8.60	10.10	8.90	2.65
Rank		4	2	1	11	6	7	8	5	9	12	10	3

#### Convergence behavior analysis

**Fig. 2:**
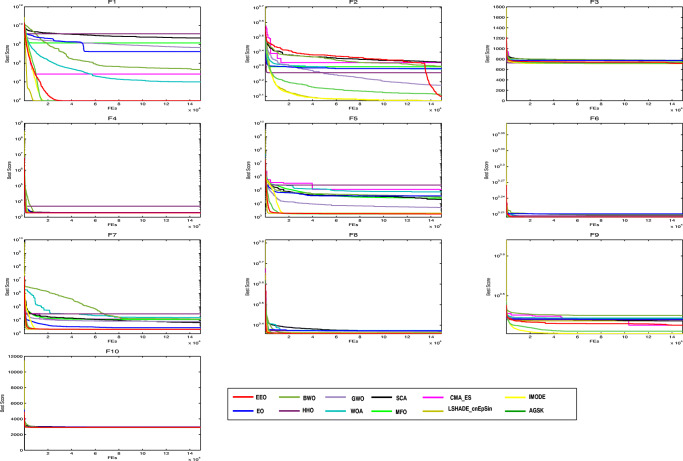
Convergence curves of the proposed EEO and the other algorithms obtained on CEC’20 test suite with *Dim* = 10

The convergence of the algorithms is evaluated (see Fig. 2. As shown in the figure, the EEO algorithm converges to (near)-optimal solutions faster than most of the other algorithms, and is therefore a viable optimization technique for problems requiring fast computation, such as online optimization.

#### Wilcoxon rank test analysis

Wilcoxon’s rank-sum test is performed to show the significance of the achieved results. Wilcoxon test demonstrates that the algorithm behavior is not random. Although, MAs are stochastic ones the probable performance is expected to be precise. For more details relating to Wilcoxon’s test, interested reader can refer to [38]. Wilcoxon’s rank sum test based on the average values is used to investigate the difference between EEO and the other optimization techniques. Table 5 compares the results obtained by EEO and the other algorithms. The “Better” column represents the sum of ranks on 10 problems when the EEO is better than the other methods. However, the “Worse” column denotes the sum of ranks on 10 functions when the EEO is worse than the other methods. The “p-value” is the significance test that decides whether the similarity hypothesis should be rejected. The significance test level should be less than 5%.

**Table 5:** Wilcoxon test (p ≥ 0.05) for the CEC’20 test suite statistical results

Compared Algorithms	Criteria	Better	Similar	Worse	p-value
EEO vs. LSHADE_cnEpSin	Average	4	5	1	0.359
EEO vs. IMODE	Average	5	3	2	1.00
EEO vs. AGSK	Average	3	5	2	0.426
EEO vs. CMA-ES	Average	9	1	0	0.002
EEO vs. MFO	Average	8	2	0	0.002
EEO vs. SCA	Average	9	1	0	0.002
EEO vs. WOA	Average	8	2	0	0.002
EEO vs. GWO	Average	8	2	0	0.002
EEO vs. HHO	Average	10	0	0	0.002
EEO vs. BWO	Average	10	0	0	0.002
EEO vs. EO	Average	10	0	0	0.002

Due to Friedman rank, EEO performs better than the MFO, SCA, WOA, GWO, HHO, BWO, and EO, in the same context, EEO compete with the CEC’20 competition winners including the IMODE and AGSK. In specific way, similar to the AGSK, IMODE and LSHADE_cnEpSin, the proposed EEO reached the optimum value for F1. For the mult-imodal F2 and F3 test methods, the proposed algorithm achieved a better performance rather than the AGSK algorithm. Over the hybrid functions F5 to F7, the EEO exhibits a robust performance near the optimal solution, i.e. the EEO and AGSK achieved the best solutions of functions F5 and F7 compared to the remain algorithms. For the composite functions F8 to F10 the EEO algorithm shows a comparative performance against the IMODE and AGSK winner algorithms. Aso, it is observed that the EEO get low performance on test functions F2 and F9, as a result the EEO get the third rank on the friedman test after AGSK and IMODE algorithms.

#### Qualitative metrics analysis

**Fig. 3:**
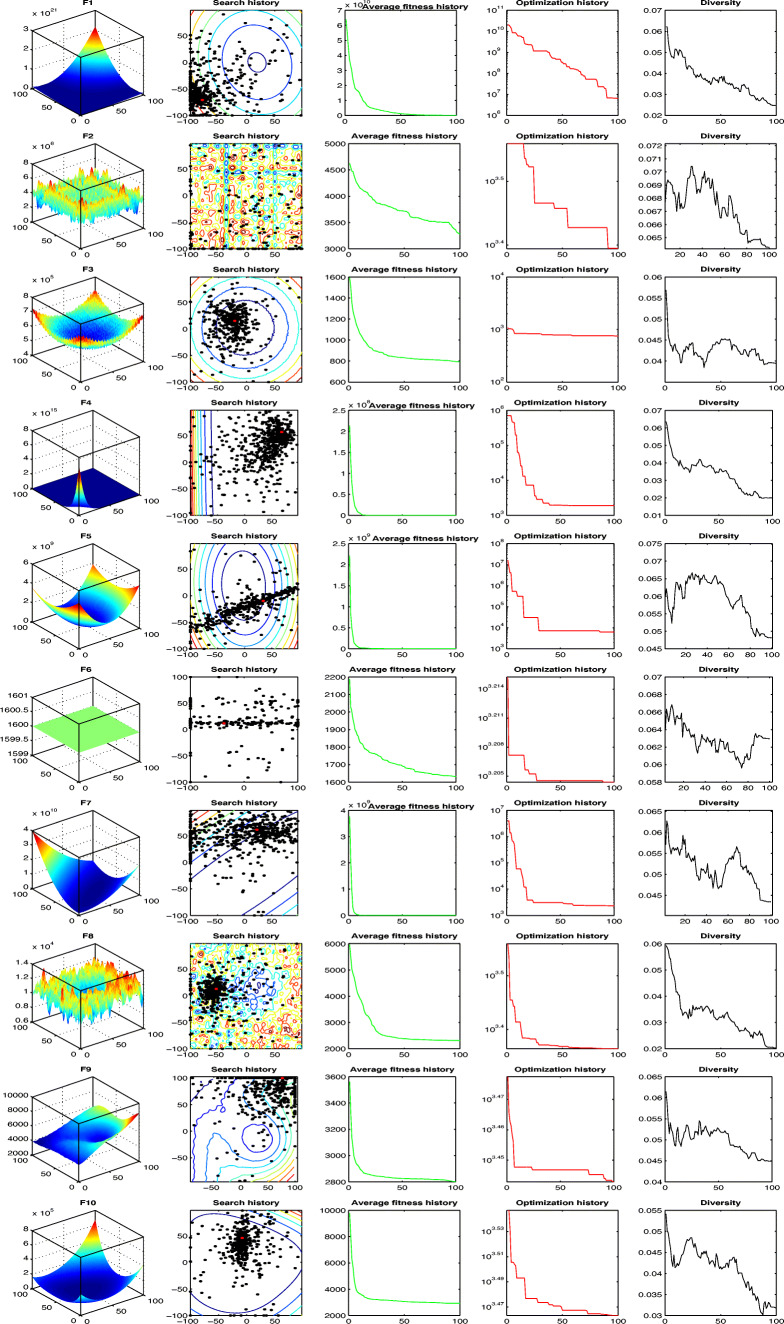
The qualitative metrics on CEC’20 test suite: 2D views of the functions, search history, average fitness history, and optimization history

The qualitative analysis of the proposed EEO algorithm are illustrated in Fig. 3. Notably, the agent’s behaviors are displayed in Fig. 3, which include 2D views of the functions, search history, average fitness history, and convergence curves. The qualitative analysis depicts the exploration/exploitation balance of the optimization algorithm, through various metrics, especially the fluctuation of solutions diversity [39, 40], over the course of the optimization process.

The following points are worthwhile from the qualitative analysis:

- *In terms of domain’s topology - functions in 2D views:* The first column of Fig. 3 depicts the 2-dimensional space of the optimization test method. Further, the test functions have a various topologies, which in turn extends a focus into determining the type of function spaces, the optimization algorithm yields a better performance.
- *In terms of regarding the search history:* The search history of the EEO particles, over the course of the iterations, is illustrated in the second column of the Fig. 3. Where the increasing of the fitness value through the search space is represented with counter lines, which in turn gradated from the blue lines to red lines with high fitness value. In this point, the Search history reveal that for some functions the EEO is able to get the areas Where the fitness values are the lowest.
- *In terms of average fitness history:* The third column of Fig. 3 explains the average fitness history, such that the averages of fitness value as a function of the iteration number. This metric concentrates the light over the general behavior of the particles through the optimization process. Particularly, the result history curves are in a decreasing pattern, which is referring the improve of particles at each iteration. This stable improvement confirms a cooperative searching behavior between the EEO particles.
- *In terms of population diversity:* The diversity plot curves are presented in the last column of Fig. 3, these curves depict the average distance between the population particles during the optimization process. The result diversity curves show that, at the iterations, the particles are most likely exploring the search space with a high diversity value. While the optimization progresses, the particles converge towards the best solution, in the exploitation phase, matched with a decreasing in the diversity value. The stable interchange in the particles diversity boost the exploration/exploitation balance strategy in the EEO algorithm.

In summary, from the results obtained, the following points can be observed:

- The proposed EEO reached the optimal value for F1, F3, F4, F6 and F7 and near-optimal value for F5, F8, and F10. These results strongly suggest that the proposed EEO could perform well on other functions with similar characteristics.
- The proposed EEO reached equivalent or better results than the other algorithms on most CEC’20 functions, as shown in Table 4.
- The Wilcoxon’s rank-sum test confirms that the EEO algorithm is statistically significant.
- The convergence curves in Fig. 2 confirm that the proposed algorithm has better exploration and exploitation abilities than the other algorithms. For most functions, some of the other algorithms either get stuck in a local optimum or fail to converge to a lower value, indicating respectively poorer exploration ability and poorer exploitation capability than those of the proposed algorithm. The improved exploitation and exploration abilities result from addition of the LF and reinforcement exploration/exploitation strategies to the original EO algorithm.

### Experimental series 2: applying EEO for solving OPF problems

**Fig. 4:**
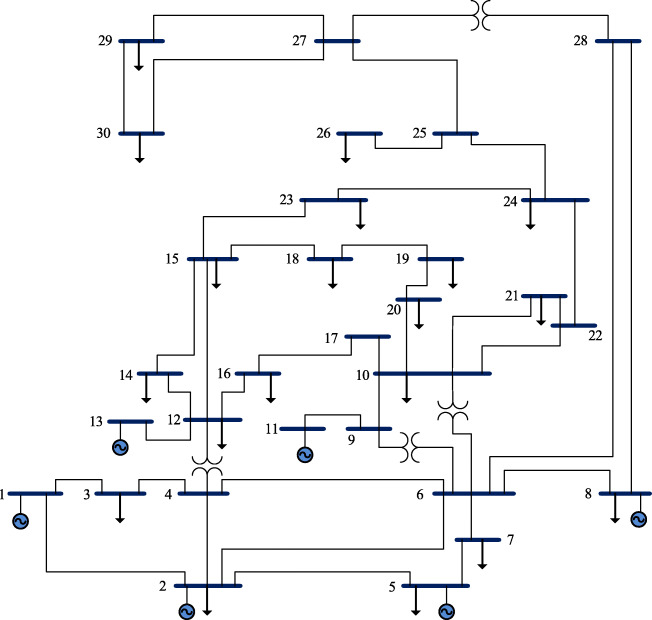
Single-line diagram of IEEE 30-bus system [42]

The OPF problem-solving ability of the proposed EEO is evaluated. For this evaluation, we compared the proposed EEO to the original EO using the standard IEEE 30-bus system; Fig. 4 shows the single-line diagram of the system [41]. Its characteristics are presented in Tables 10, 11 and 12 in the Appendix. This system has 24 control variables which consist of the active power of PV buses, voltages magnitudes of generator buses, transformer ratio, and shunt reactive power compensating. Furthermore, the transformer tap and shunt reactive power compensating among the control variables both are discrete variables.

We performed 20 independent runs for each objective function detailed in subsection 4.2; Table 6 lists the six case studies. We run the simulations with MATLAB R2016a on a computer that has a 2.4 GHz processor and 8 GB RAM. For both algorithms, the maximum number of iterations is 300 and the population size is 30.

**Table 6:** Different objectives that will be minimized for solution of the OPF problem

Case no.	Objective function to minimize
1	Fuel cost
2	Fuel cost with value-point loading effect
3	Total emission
4	Total active power loss (Ploss)
5	Voltage instability (L-index)
6	Voltage deviation (VD)

Table 7 presents the settings of all control and state (dependent) variables along with their allowable ranges for the best fitness value obtained for the objective function in a case study pertaining to a 30-bus test system using the proposed EEO and original EO algorithms. Active power of swing generator (PG1) and reactive power of all generators are states or dependent variables treated as constraints in the optimization. Values of these variables are listed here to show that the proposed EEO techniques duly comply with the limits of these constraining variables for the six cases. The statistical results of 20 runs for each study case performed (i.e. case 1 to case 6) using the EEO and EO algorithms are presented in Table 8. The columns indicate the best, mean, worst, and standard deviation (STD) values for the objective function in each case. The best solutions are shown in boldface.

**Table 7:** Optimized solutions obtained by the proposed EEO and the original EO

Parameters	Mi	Max	Case 1		Case 2		Case 3		Case 4		Case 5		Case 6	
Algorithm			EEO	EO	EEO	EO	EEO	EO	EEO	EO	EEO	EO	EEO	EO
PG1(MW)	50	200	176.7345	177.1761	198.8185	198.4615	64.03941	63.94042	51.49013	51.48659	69.56936	100.0961	129.6951	179.2273
PG2(MW)	20	80	48.78826	48.4943	45.57374	44.74133	67.58938	67.6997	79.99994	79.99965	79.83622	71.84979	47.32925	57.44291
PG5(MW)	15	50	21.35174	20.9208	17.72303	18.738	50	50	49.99974	49.99994	49.82619	47.22927	49.09913	15.00639
PG8(MW)	10	35	21.59308	21.4246	10.01422	10.01188	35	35	34.99917	35	33.28169	19.60833	35	20.64538
PG11(MW)	10	30	11.90222	12.3835	10.00262	10.00616	29.99947	30	30	30	29.98874	20.46415	12.8704	10
PG13(MW)	12	40	12.01063	12.0122	12.00087	12.06352	39.99948	40	39.99999	39.99956	24.53242	29.13496	15.71997	12.02821
V1 (p.u.)	0.95	1.10	1.081652	1.0819	1.082469	1.085627	1.060609	1.057223	1.061175	1.062489	1.067295	1.062388	1.0139	1.027393
V2 (p.u.)	0.95	1.10	1.083692	1.08287	1.099987	1.062635	1.096574	1.072947	1.057169	1.099967	1.045981	1.053533	0.996499	1.016143
V5 (p.u.)	0.95	1.10	1.034161	1.03214	1.025156	1.084539	1.03463	1.030734	1.037851	1.040228	1.08988	1.098498	1.06786	1.069078
V8 (p.u.)	0.95	1.10	1.040193	1.03681	1.032488	1.035779	1.042812	1.038508	1.043643	1.047144	1.044445	1.011257	0.999616	1.004705
V11 (p.u.)	0.95	1.10	1.058904	1.07112	1.083962	1.064525	1.064226	1.050502	1.050452	1.048088	1.06065	1.098905	1.069644	1.002025
V13 (p.u.)	0.95	1.10	1.035318	1.03504	1.060129	1.048758	1.054932	1.052189	1.051736	1.02486	0.986456	1.054714	0.95	0.993956
Qc10(MVAr)	0.0	5.0	1.039025	1.04613	1.061527	1.008772	0.992369	1.024277	1.02419	1.053901	1.065474	1.087365	1.091928	1.012106
Qc12(MVAr)	0.0	5.0	0.9264	0.90434	0.906406	0.967251	0.996164	0.906325	0.933052	0.902755	0.903663	0.902645	0.900017	0.900699
Qc15(MVAr)	0.0	5.0	0.963273	0.97168	0.99077	0.966531	0.98598	0.992144	0.990881	0.972466	0.970709	1.006587	0.963644	0.972933
Qc17(MVAr)	0.0	5.0	0.971492	0.97228	0.971138	0.981924	0.971039	0.958447	0.973498	0.975594	0.987518	0.981771	0.971139	0.969432
Qc20(MVAr)	0.0	5.0	0.045018	0.04487	8.53E-05	0.047422	0.04992	0.012074	0.049819	0.043753	0.04994	0.049442	0.048274	0.041702
Qc21(MVAr)	0.0	5.0	0	0.04019	0	0.004389	0.012866	0.049578	0.03386	0.002621	0.05	0.049951	0.043937	0.049475
Qc23(MVAr)	0.0	5.0	0.049426	0.04851	0.049003	0.049036	0.048544	0.049848	0.049166	0.047334	0.049857	0.049603	0.049727	0.043814
Qc24(MVAr)	0.0	5.0	0.048943	0.00575	0.049549	0.049962	0.049001	0.046831	0.049966	0.049064	0.049987	0.049425	3E-08	9.42E-05
Qc29(MVAr)	0.0	5.0	0.043546	0.04348	0.041962	0.046633	0.046291	0.049417	0.039794	0.05	0.049996	0.048644	0.05	0.049961
T11 (p.u.)	0.90	1.10	0.049801	0.04985	0.049802	0.045002	0.049972	0.049913	0.049996	0.049946	0.049588	0.049935	0.049891	0.049994
T12 (p.u.)	0.90	1.10	0.035215	0.03513	0.05	0.017566	0.005944	0.03156	0.029683	0.029382	0.049999	0.049619	0.049769	0.049726
T15 (p.u.)	0.90	1.10	0.04999	0.04915	0.021882	0.049955	0.049434	0.049906	0.05	0.05	0.05	0.049921	0.049795	0.049973
T36 (p.u.)	0.90	1.10	9.03E-05	0.01994	0.019325	0.044025	0.021643	0.000267	0.018962	0.017501	0.049985	0.049511	0.027406	0.02472
Fuel cost ($/h)	**–**	**–**	**800.4145**	800.4334	804.8165	804.3562	944.3474	944.5611	967.5845	967.5772	931.9871	887.8918	864.2726	809.101
FC VPE($/h)	**–**	**–**	843.1672	843.057	**832.1817**	832.1969	1015.448	1015.589	1027.333	1027.324	996.604	952.6482	909.6526	848.4336
Emission(t/h)	**–**	**–**	0.365163	0.366289	0.438335	0.43666	**0.204821**	0.204825	0.207265	0.207265	0.215376	0.233406	0.266382	0.375725
Ploss (MW)	**–**	**–**	8.99217	9.023216	10.74492	10.62788	3.238253	3.250641	**3.088974**	3.096271	3.634622	4.988621	6.313853	10.95594
L-index (max)	**–**	**–**	0.129852	0.127399	0.129291	0.126421	0.127141	0.128741	0.126786	0.127273	**0.124236**	0.124268	0.136279	0.136635
VD (p.u.)			0.870319	0.910383	0.841403	0.884285	0.896563	0.894562	0.913766	0.915076	0.980597	0.974147	**0.089035**	0.090567
QG1 (MVAr)	-20	150	-0.00299	0.016493	0.033433	0.064999	-0.09184	-0.08636	-0.05728	-0.0762	0.065898	-0.0904	-0.19987	-0.19653
QG2 (MVAr)	-20	60	0.068015	0.056597	0.024276	0.006368	0.00397	0.010094	-0.05432	-0.00088	-0.26341	-0.17932	-0.1626	-0.03944
QG5 (MVAr)	-15	62.5	0.073824	0.06759	0.03361	0.103348	0.001255	0.008086	0.028024	0.027586	0.150403	0.180893	0.314862	0.411097
QG8 (MVAr)	-15	48.7	-0.00652	-0.0607	-0.08965	-0.05022	-0.05801	-0.02562	-0.05241	-0.007	-0.08544	-0.29114	-0.07664	0.136455
QG11(MVAr)	-10	40	0.113771	0.167271	0.250877	0.102016	0.081167	0.071331	0.064693	0.090136	0.145719	0.324483	0.359756	0.006851
QG13(MVAr)	-15	44.7	-0.0338	-0.03611	0.086157	-0.00831	0.047388	0.026594	0.023187	-0.02814	-0.09366	0.045614	-0.06573	-0.03732

**Table 8:** Statistical analysis of the proposed EEO and the original EO

Case no.	Method	Best	Mean	Worst	STD
Case 1	EEO	**800.4145068**	**800.5418237**	**800.6858073**	**0.082200045**
	EO	800.4333827	800.566219	800.8858013	0.147686575
Case 2	EEO	**832.1817334**	**832.6177702**	**833.5261004**	**0.305491785**
	EO	832.1969006	832.6990767	834.1425238	0.484922433
Case 3	EEO	**0.2048212**	**0.20484045**	**0.2048727**	**1.65853E-05**
	EO	0.20482522	0.2048702	0.2049223	3.09025E-05
Case 4	EEO	**3.088973939**	**3.129399194**	**3.176506296**	**0.025197238**
	EO	3.096270703	3.136782048	3.191664999	0.025250504
Case 5	EEO	**0.124235794**	**0.125074084**	**0.125744463**	**0.000437337**
	EO	0.124268107	0.125272548	0.127181735	0.000762836
Case 6	EEO	**0.089034887**	**0.095279721**	**0.099550829**	**0.003002618**
	EO	0.090567 289	0.098106481	0.117290411	0.00629304

**Fig. 5:**
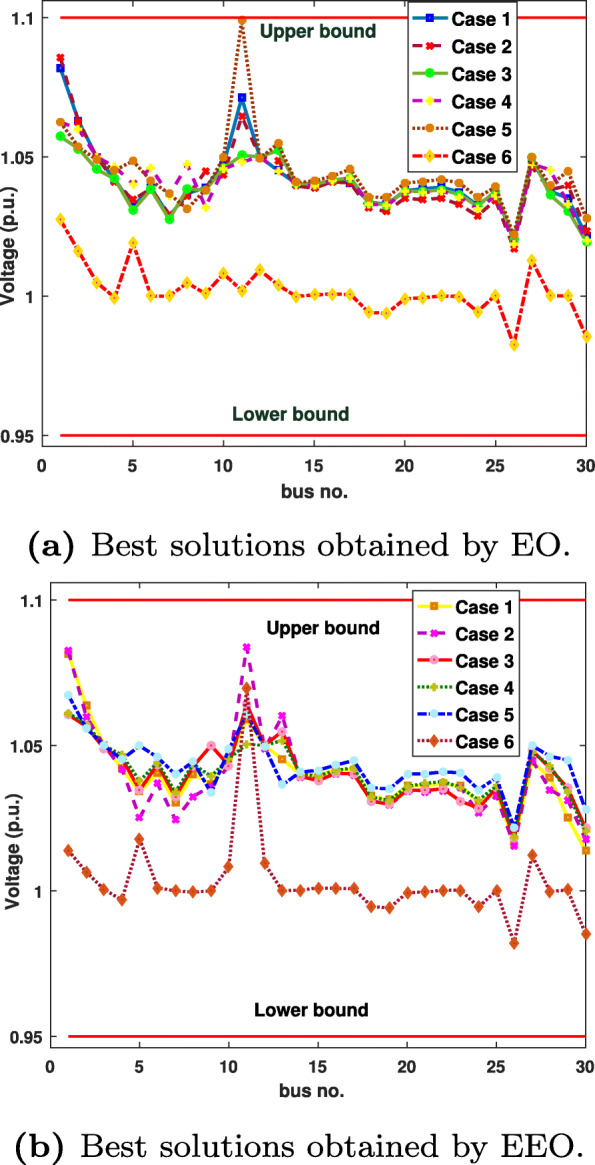
Voltage profiles of the load buses

For all objective functions, the proposed algorithm reached better solutions in regards to the average, best, worst, and STD values. Figure 5 presents the voltage profiles of the load buses obtained by EO and EEO for the six cases.

**Fig. 6:**
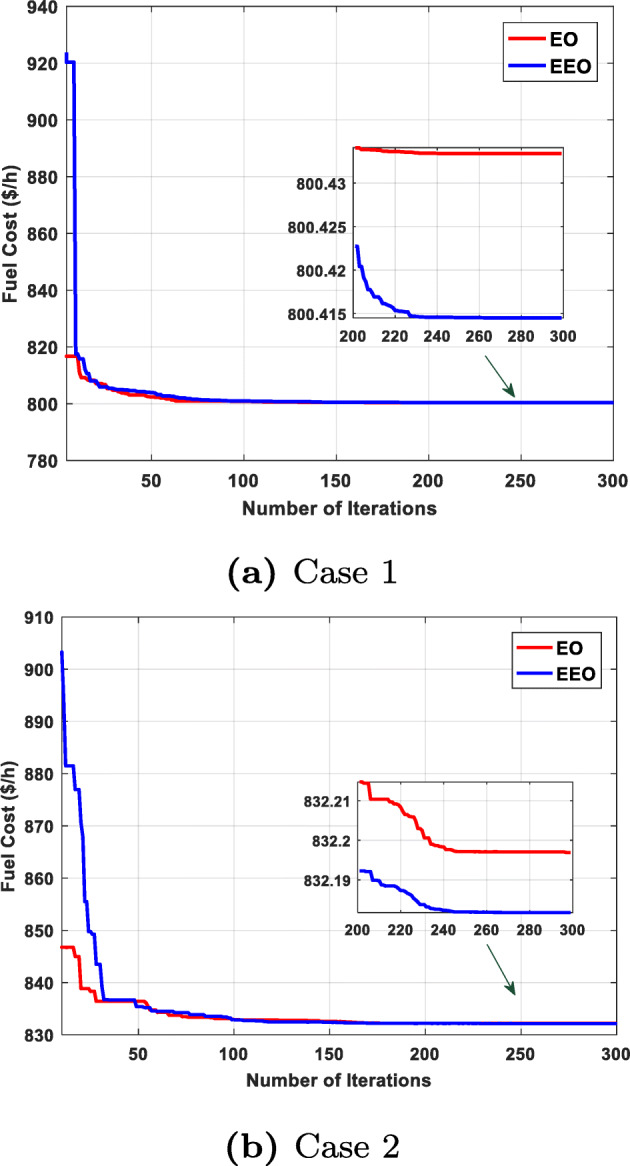
Convergence curves

**Fig. 7:**
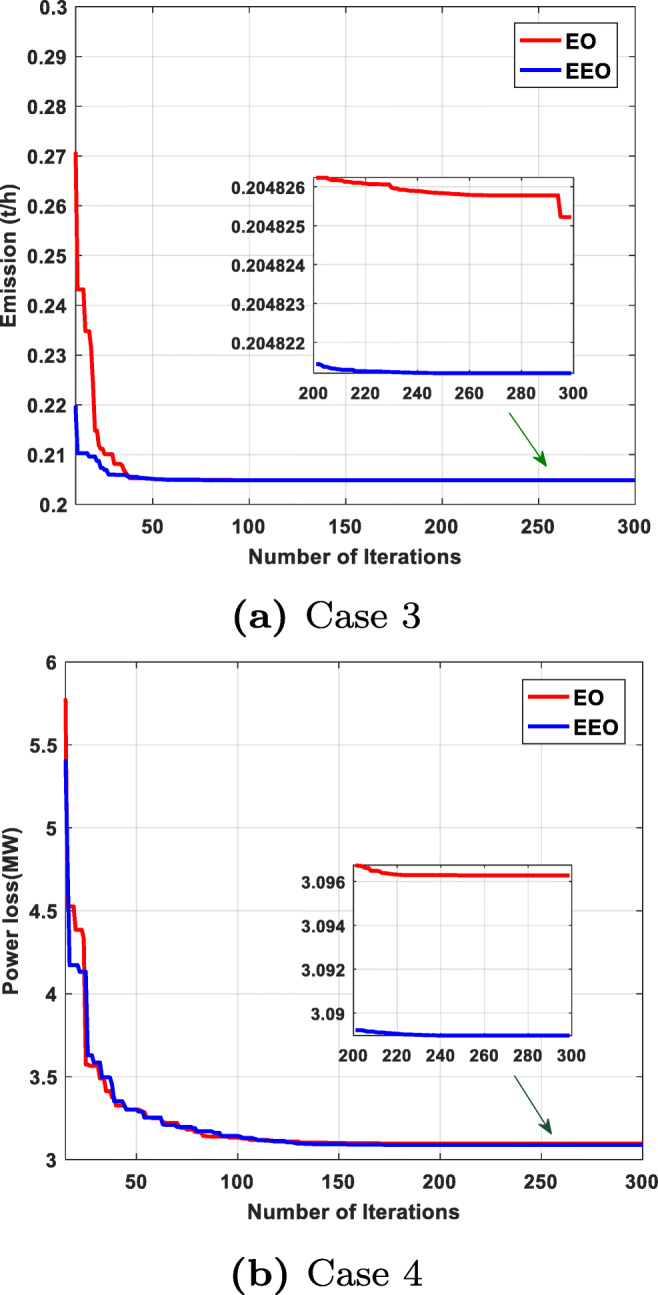
Convergence curves

**Fig. 8:**
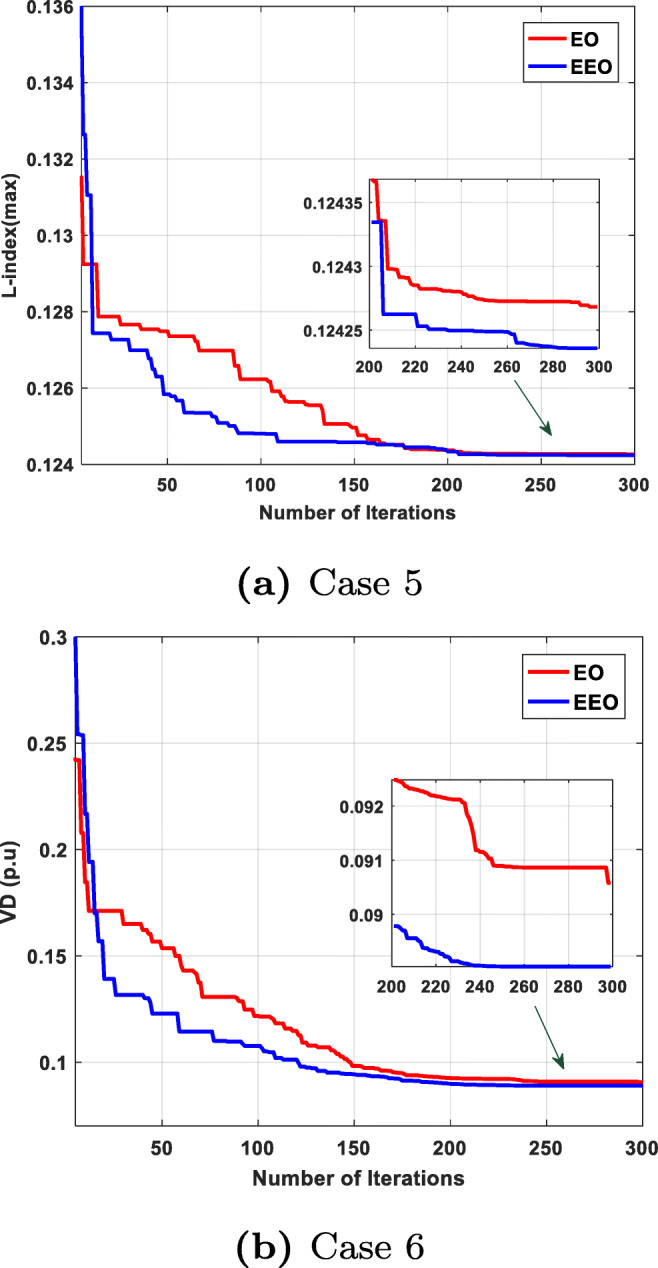
Convergence curves

**Fig. 9:**
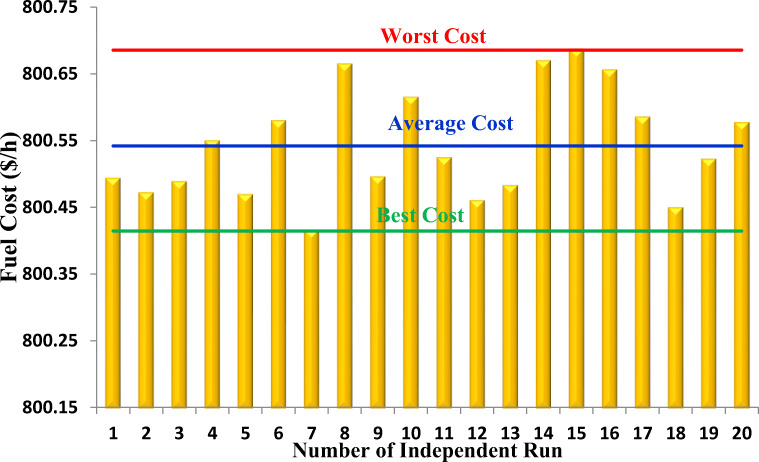
Data distribution obtained by EEO - Case 1

We can see that EEO yields voltages within the lower and upper bounds. The safety margin (the largest absolute value of the voltage difference between the bounds and the value obtained by the algorithm) values achieved with EEO are higher than those achieved with EO. Thus, the optimized system by EEO will tolerate higher voltage perturbations than the system optimized by the original EO. Figures 6 to 8 compare the convergence curves of both algorithms; except for case 3, in the beginning, the original EO has lower objective function values than EEO, but after fewer than 50 iterations, the proposed EEO reaches lower values. Narrow data distributions are obtained for all cases (highest STD value obtained by EEO: 0.305491785). Figure 9 shows the 20 independent run distributions obtained by the proposed EEO for Case 1.

Fuel cost minimization is also performed. The EEO obtained a fuel cost of 800.415 $/h, which is lower than the value obtained by the original EO (800.433 $/h) and other techniques, as shown in Table 9. Figure 6a compares the convergence characteristics of EEO and EO; the EEO performs better than the EO. Table 7 presents the statistical analysis of the optimized solutions. In addition, Figs 6b and 7a show the load bus voltages of the IEEE 30-bus system for the cases considered.

**Table 9:** Comparison of EEO and other studied optimization algorithms

Cases	Algorithms	Fuel cost ($/h)	Emission (t/h)	Ploss (MW)	VD (p.u.)	L-index (max)
Case 1	EEO	**800.4145**	0.365163	8.99217	0.870319	0.129852
	EO	800.4334	0.366289	9.023216	0.910383	0.127399
	MGOA [5]	800.4744	0.3649	8.9882	0.8851	0.1295
	FCGCS [43]	800.4173	**–**	9.0127	0.9131	0.1376
	DGWO [44]	800.433	**–**	8.6428	0.7285	0.1299
	MSA [8]	800.5099	0.36645	9.0345	0.90357	0.13833
	AGSO [45]	801.75	0.3703	**–**	**–**	**–**
	Jaya [46]	800.4794	**–**	9.06481	**–**	0.1273
	ABC [47]	800.66	–	9.0328	**–**	0.1381
	SKH [48]	800.5141	0.3662	9.0282	**–**	0.1382
	BSA [49]	799.0760^a^	0.3671	8.6543	1.9129^a^	0.1273
	PSOGSA [50]	800.49859	**–**	9.0339	**–**	0.12674
	ICBO [10]	799.0353^a^	**–**	8.6132	1.9652^a^	0.1261
	SCA [7]	800.1018^a^	**–**	9.0633	**–**	**–**
	APFPA [51]	798.9144^a^	**–**	8.5800	1.9451^a^	**–**
	FHSA [49]	799.914^a^	**–**	**–**	1.5265^a^	–
	GEM [52]	799.0463^a^	0.3665	8.6257	1.9312^a^	0.1264
	DE [53]	799.0827^a^	**–**	8.63	1.8505^a^	0.1277
Case 2	EEO	**832.1817**	0.438335	10.74492	0.841403	0.129291
	EO	832.1969	0.43666	10.62788	0.841403	0.126421
	PSO [10]	832.6871	**–**	**–**	**–**	**–**
	ICBO [10]	830.4531^a^	**–**	10.2370	1.7450^a^	0.1289
	BSA [49]	830.7779^a^	0.4377	10.2908	1.2050^a^	0.1363
	APFPA [51]	830.4065^a^	**–**	10.2178	1.8909^a^	**–**
Case 3	EEO	944.3474	**0.204821**	3.238253	0.896563	0.127141
	EO	944.5611	0.204825	3.250641	0.894562	0.128741
	MSA [8]	944.5003	0.20482	3.2358	0.87393	0.13888
	DSA [54]	944.4086	0.20583	3.2437	**–**	0.12734
	AGSO [45]	953.629	0.2059	**–**	**–**	**–**
	GEM [52]	943.6358^a^	0.2048^a^	3.0160	1.9504^a^	0.1269
Case 4	EEO	967.5845	0.207265	**3.088974**	0.913766	0.126786
	EO	967.5772	0.207265	3.096271	0.915076	0.127273
	MSA [8]	967.6636	0.20727	3.1005	0.88868	0.13858
	DSA [54]	967.6493	0.20826	3.0945	**–**	0.12604
	EM [9]	954.3150	**–**	3.1775	**–**	0.1253
	IEM [9]	967.1147	**–**	2.8699^a^	**–**	0.1156
	APFPA [51]	965.6590^a^	**–**	2.8463^a^	2.0720^a^	–
	ABC [47]	967.681	**–**	3.1078	**–**	0.1386
	GEM [52]	966.7473^a^	0.2072	2.8863^a^	1.9755^a^	0.1265
Case 5	EEO	931.9871	0.215376	3.634622	0.980597	**0.124236**
	EO	887.8918	0.233406	4.988621	0.974147	0.124268
	ECHT-DE [55]	917.5916	**–**	4.5224	**–**	0.13632
	SEUMRE [56]	918.1040	**–**	3.3194	**–**	0.0769^a^
	SSO [57]	**–**	**–**	**–**	**–**	0.1267
	NISSO [57]	**–**	**–**	**–**	**–**	0.12547
Case 6	EEO	864.2726	0.266382	6.313853	**0.089035**	0.136279
	EO	809.101	0.375725	10.95594	0.090567	0.136635
	SKH [48]	814.0100	0.3740	9.9056	**–**	0.1366
	GEM [52]	816.9095^a^	0.2802	6.2313	1.8320^a^	0.1257
	DE [53]	915.2172	**–**	3.626	2.1064^a^	0.1243
	HS [58]	895.6223	**–**	4.3244	0.1006	**–**
	Jaya [46]	840.7181	**–**	7.884	0.1243	**–**
	MSCA [7]	849.2812	**–**	7.0828	0.1031	**–**

^a^ The constraint on load bus voltage is not respected, making this an impractical option

#### Case 2: minimizing the fuel cost with value-point loading effect

This case aims to minimize the fuel cost associated with the value-point loading effect. Figure 6b shows the convergence of the studied algorithms; the EEO outperforms the original EO in terms of achieving the best solution. Table 7 shows that total fuel costs of 832.1817 $/h and 832.1969 $/h are achieved for EEO and EO, respectively, indicating the superiority of the proposed EEO to the original EO. Table 9 compares the best values of the costs obtained by the EEO with other counterparts.

#### Case 3: minimizing emission

This case is aimed at minimizing the total emission, thereby reducing pollution. The results obtained for this case are provided in Table 7, which reveals that EEO and EO achieve optimal total emissions of 0.2048212 t/h and 0.20482522 t/h, respectively. Figure 7a shows the convergence curves obtained by both algorithms. Furthermore, Table 9 compares the best result obtained by the proposed algorithm with the results available in the literature; the EEO provides one of the best results.

#### Case 4: minimizing total active power loss

In this case, the OPF solutions are optimized by considering the optimal total active power loss values. The convergence characteristics of the considered algorithms for this case are shown in Fig. 7b. From Table 9, the optimal power losses obtained by the EEO and EO are 3.088974 MW and 3.096271 MW, respectively. Compared with recent techniques (see Table 9), the EEO provides an approved solution and guarantees that all limits are always respected, unlike some of the published algorithms.

#### Case 5: voltage stability enhancement

The main objective of this case is to obtain the best values of voltage stability enhancement. The optimal settings of design variables for the finest stability enhancement by EEO and basic EO are listed in Table 7. As shown in the table, the L-index (the voltage stability indicator) obtained by EEO, 0.124236, is lower and therefore better than the value (0.124268) obtained by the original EO. A comparison of the EEO and EO convergence characteristics (Fig. 8a) reveals that the EEO outperforms the original EO in terms of convergence rate and optimized solution.

#### Case 6: minimizing the voltage deviation

The obtained results from the proposed algorithm and original algorithms for case 6 are listed in Table 7. Table 9 presents the results obtained by the proposed technique and other optimization techniques used for solving the same case. We see from the tables that, compared with the other algorithms, the proposed algorithm achieved a better solution. The convergence curves obtained from the proposed EEO and the original EO of case 6 are presented in Fig. 8b; the proposed method yields rapid convergence to the best solution.

#### Comparing EEO with published studies

We compared the EEO results to more than 20 published results as shown in Table 9. Compared with other methods, the EEO achieved lower function values in most cases, substantiating the EEO efficiency. Thus, at a large scale, substantial cost savings and emission reductions can potentially be achieved by EEO while improving the system stability.

## Conclusion and future work

This paper proposed an enhanced version of the Equilibrium Optimizer (EO) called EEO, which relies mainly on reinforcing the algorithm exploration and exploitation process. The proposed EEO is applied for obtaining improved solutions to global optimization problems, and Optimal Power Flow (OPF) problems. During the process, we assess the performance of the proposed EEO with regard to ten functions of the CEC’20 test suite. The EEO achieved better or similar results than LSHADE_cnEpSin, CMA-ES, IMODE, AGSK, MFO, SCA, WOA, GWO, HHO, BWO, and EO. Wilcoxon’s rank-sum test confirms that the proposed EEO results are statistically significant. Moreover, we demonstrated the efficiency of the proposed EEO on OPF for the standard IEEE 30-bus system. We minimized different objectives, i.e., the fuel cost, fuel cost with value-point loading effect, total emission, active power loss, voltage deviation, and voltage instability. For most objectives, the EEO yielded better results than the original EO and the methods reported in 20 published studies. Substantial cost savings and emission reductions can potentially be achieved with EEO at a large scale while improving system stability. Thus, the proposed algorithm is a valuable optimization tool for engineers of power systems and a promising tool for solving more complex optimization problems than those associated with such systems.

Indeed, as future work, the proposed EEO will be applied to more challenging problems (than the problem considered here), including multi-objective problems and problems such as prediction, image segmentation, Cloud Data Center [59, 60], and prediction of cloud workloads [61–63].

## Appendix A: Data of IEEE 30-bus test system

Table 10 presents as in [5] the characteristics if the IEEE 30-bus system.

**Table 10:** Characteristics of IEEE 30-bus test system

Characteristics	Value details	Details
Buses	30	11
Branches	41	–
Generators	6	Buses: 1, 2, 5, 8, 11 and 13
Load voltage limits	24	[0.95:1.05]
Shunt VAR compensation	9	Buses: 10, 12, 15, 17, 20, 21, 23, 24 and 29.
Transformers	4	Branches: 11, 12, 15 and 36
Control variables	24	–

Table 11 presents as in [64] the branch data used for the IEEE 30-bus system.

**Table 11:** Branch data used for IEEE 30-bus system

Branch no.	Bus no.		R (p.u.)	X (p.u.)	B/2 (p.u.)	Rating (MVA)	Branch no.	Bus no.		R (p.u.)	X (p.u.)	B/2 (p.u.)	Rating (MVA)
	From	To						From	To				
1	1	2	0.0192	0.0575	0.0264	130	22	15	18	0.1073	0.2185	0	16
2	1	3	0.0452	0.1652	0.0204	130	23	18	19	0.0639	0.1292	0	16
3	2	4	0.0570	0.1737	0.0184	65	24	19	20	0.0340	0.0680	0	32
4	3	4	0.0132	0.0379	0.0042	130	25	10	20	0.0936	0.2090	0	32
5	2	5	0.0472	0.1983	0.0209	130	26	10	17	0.0324	0.0845	0	32
6	2	6	0.0581	0.1763	0.0187	65	27	10	21	0.0348	0.0749	0	32
7	4	6	0.0119	0.0414	0.0045	90	28	10	22	0.0727	0.1499	0	32
8	5	7	0.0460	0.1160	0.0102	70	29	21	22	0.0116	0.0236	0	32
9	6	7	0.0267	0.0820	0.0085	130	30	15	23	0.1000	0.2020	0	16
10	6	8	0.0120	0.0420	0.0045	32	31	22	24	0.1150	0.1790	0	16
11	6	9	0	0.2080	0	65	32	23	24	0.1320	0.2700	0	16
12	6	10	0	0.5560	0	32	33	24	25	0.1885	0.3292	0	16
13	9	11	0	0.2080	0	65	34	25	26	0.2544	0.3800	0	16
14	9	10	0	0.1100	0	65	35	25	27	0.1093	0.2087	0	16
15	4	12	0	0.2560	0	65	36	28	27	0	0.3960	0	65
16	12	13	0	0.1400	0	65	37	27	29	0.2198	0.4153	0	16
17	12	14	0.1231	0.2559	0	32	38	27	30	0.3202	0.6027	0	16
18	12	15	0.0662	0.1304	0	32	39	29	30	0.2399	0.4533	0	16
19	12	16	0.0945	0.1987	0	32	40	8	28	0.0636	0.2000	0.0214	32
20	14	15	0.2210	0.1997	0	16	41	6	28	0.0169	0.0599	0.0065	32
21	16	17	0.0524	0.1923	0	16							

Table 12 presents as in [64] the branch data used for the IEEE 30-bus system.

**Table 12:** Cost and emission coefficients of generators for IEEE 30-bus system

Generator	Bus	a	b	c	d	e	*α*	*β*	*σ*	*γ*	*μ*
$P_{G_{1}}$	1	0	2	0.00375	18	0.037	4.091	-5.554	6.49	0.0002	2.857
$P_{G_{2}}$	2	0	1.75	0.0175	16	0.038	2.543	-6.047	5.638	0.0005	3.333
$P_{G_{3}}$	5	0	1	0.0625	14	0.04	4.258	-5.094	4.586	0.000001	8
$P_{G_{4}}$	8	0	3.25	0.00834	12	0.045	5.326	-3.55	3.38	0.002	2
$P_{G_{5}}$	11	0	3	0.025	13	0.042	4.258	-5.094	4.586	0.000001	8
$P_{G_{6}}$	13	0	3	0.025	13.5	0.041	6.131	-5.555	5.151	0.00001	6.667
